# A Case of a Persistent Postoperative Infection Caused by Multidrug-Resistant *Kluyvera ascorbata* in the Oral and Maxillofacial Region

**DOI:** 10.1155/2019/2180567

**Published:** 2019-01-31

**Authors:** Si-Hai Zou, Lu-Ying Zhu, Yong Li, Fu-Gui Zhang

**Affiliations:** ^1^Department of Oral and Maxillofacial Surgery, Affiliated Hospital of Stomatology, Chongqing Medical University, Chongqing 401147, China; ^2^Chongqing Key Laboratory of Oral Diseases and Biomedical Sciences, Chongqing 401147, China

## Abstract

**Introduction:**

*Kluyvera ascorbata* infection is rare, but it has been extensively studied because of its potential to cause a wide range of infections and its ability to transfer the gene encoding for CTX-M-type extended spectrum *β*-lactamases (ESBLs) to other Enterobacteriaceae.

**Case Presentation:**

The authors report a case of a 61-year-old Chinese male with a persistent postoperative infection caused by a multidrug-resistant ESBL-producing *K. ascorbata*. Following antimicrobial susceptibility testing, he was aggressively treated with gentamicin and levofloxacin with a favorable outcome.

**Conclusion:**

To our knowledge, this is the first case report of a persistent postoperative infection caused by a multidrug-resistant *K. ascorbata* in the oral and maxillofacial region. The authors suggest that *K. ascorbata* infection warrants prompt identification and aggressive antibiotic management, given that ESBL-producing *K. ascorbata* is resistant to penicillins and first-generation to third-generation cephalosporins.

## 1. Introduction

*Kluyvera ascorbata* is a Gram-negative microorganism belonging to the family of Enterobacteriaceae and was first identified by Farmer et al. [1]. Although it infrequently causes infections, it can cause a wide range of infections including acute appendicitis [2]; biliary tract infection [3]; urinary tract infection [4]; bacteremia with neutropenia and fever [5]; bacteremia and severe sepsis [6]; sepsis accompanied with multiorgan dysfunction [7]; hock and pulmonary hemorrhage [8]; enteritis, central venous catheter infections, and peritonitis [9, 10]; solid organ transplant recipient infection [11]; acute cholecystitis [12]; and cholecystitis and bacteremia [13]. However, no *K. ascorbata* infections have been reported to date in the oral and maxillofacial region. Therefore, the authors describe the first case of a persistent postoperative infection caused by *K. ascorbata* in the oral and maxillofacial region after a combination of radical neck dissection with gingivectomy and mandibulectomy. A brief literature review of the clinical features of *K. ascorbata* infections in humans is also included.

## 2. Case Presentation

**Figure 1:**
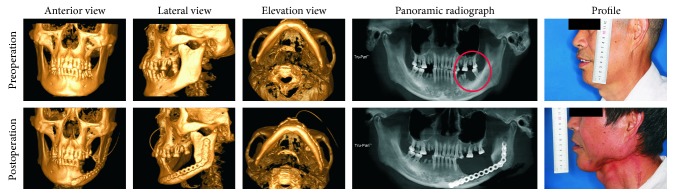
Images taken before surgery and two months after surgery. The circle shows the squamous cell carcinoma lesion of the gingiva in the left mandible. It took two months for the wound to heal after the *Kluyvera ascorbata* infection.

A 61-year-old Chinese man was admitted to hospital with a gingival squamous cell carcinoma of the left mandible. He was treated with a combination of radical neck dissection with gingivectomy, mandibulectomy, and strengthening of the mandible with a reconstructive plate (Figure 1). *K. ascorbata* was identified from the drainage specimen taken on postoperative day five and confirmed with the Hefei Star HX-21 blood culture analyzer (Hefei Star Technology Development Co., Ltd., Anhui, China). Antimicrobial susceptibility testing showed resistance to cefazolin and piperacillin but susceptibility to levofloxacin and gentamicin (Table 1). *K. ascorbata*'s ability to produce ESBLs was also detected by the same system. The patient's blood culture was sterile. Intravenous administration of levofloxacin (200 mg, q24 h) and gentamicin (240 mg, q24 h) based on the susceptibility test of this microorganism was continued for 14 days. The wounds were continuously dressed twice a day for 2 weeks and daily for 1 week. The patient was discharged home with an iodoform sponge which was changed weekly for 1 month, and the wound gradually healed after 2 months.

## 3. Discussion

*K. ascorbata* is a relatively newly described member of the Enterobacteriaceae family that rarely causes infections in humans. These bacteria are usually considered a commensal [8]. Nosocomial infections pose significant threats to hospitalized patients, especially immunocompromised patients, such as those with cancer [14]. The authors report what they believe to be the first case of a multidrug-resistant *K. ascorbata* isolated from the wound drainage specimen of an inpatient with a postoperative infection.

*K. ascorbata* is virulent in terms of clinical features. It causes a wide range of infections over a wide age span, namely, from adults as old as 78 years [15] to children [9], infants [16], low birth weight infants [8], and neonates as young as five days old [17]. The authors report a case involving a 61-year-old patient.

*Kluyvera* species can be isolated from sputum, urine, stools, and blood [18, 19], hospital sewage [20], human milk samples [21], as well as wound drainage specimens as in our case.

*Kluyvera* strains are rare but potentially dangerous pathogens due to their ability to transfer the gene encoding for ESBLs [18], which was elucidated by Literacka et al. [22]. CTX-M beta-lactamases, which are plasmids mediated in other Enterobacteriaceae, originate from the chromosomal beta-lactamases of its reservoir, *K. ascorbata*. IS*Ecp1B*, which is an insertion element [23] and a genetic mobile element [24], has been reported to be associated with gene transfer [25, 26]. Besides *bla*_CTX-M-3_, *K. ascorbata* also bears the *bla*_TEM-1_, *aacC2*, and *armA* genes, as well as integronic *aadA2*, *dfrA12*, and *sul1*, which together confer resistance to the majority of beta-lactams, aminoglycosides, and trimethoprim-sulfamethoxazoles [27]. Antimicrobial agents active against *Kluyvera* strains include third-generation cephalosporins, fluoroquinolones, aminoglycosides [8], beta-lactams with beta-lactamase inhibitors and carbapenems [7], and meropenem [26]; however, as shown by the authors, ESBL-producing *K. ascorbata* is resistant to penicillins and first-generation to third-generation cephalosporins. Clinicians should be aware of the spectrum of disease and the increasing clinical importance of this pathogen. Purified KLUA-9 from *K. ascorbata* showed the highest catalytic efficacy towards benzylpenicillin, ampicillin, piperacillin, first-generation cephalosporins, cefuroxime, and cefoperazone and the lowest efficacy towards dicloxacillin, cefoxitin, and imipenem [28].

*K. ascorbata*, which is a potentially dangerous pathogen in both immunocompetent and immunocompromised hosts, warrants prompt identification by antimicrobial susceptibility testing and a correct and aggressive antibiotic management [16, 29].

## Figures and Tables

**Table 1:** Results of antimicrobial susceptibility testing (AST).

Name	Group	MIC (*μ*g/ml)	AST
Cefazolin	A	≥8	Resistant
Ampicillin	A	≥32	Resistant
Gentamicin	A	≤2	Sensitive
PIZ	B	≥128/4	Resistant
Piperacillin	B	≥128	Resistant
Cefuroxime	B	≤4	Resistant
Cefotaxime	B	≥64	Resistant
Cefepime	B	≤4	Sensitive
Imipenem	B	≤1	Sensitive
Amikacin	B	≤8	Sensitive
Ciprofloxacin	B	≤0.25	Sensitive
Levofloxacin	B	≤1	Sensitive
SXT	B	≤0.5/9.5	Sensitive
Aztreonam	C	≥16	Resistant
Ceftazidime	C	≤4	Resistant
Chloramphenicol	C	≤4	Sensitive
Cefoperazone	O	≤8	Sensitive
Minocycline	O	≤2	Sensitive
CPS	O	≥64/64	Resistant
Nitrofurantoin	U	≥128	Resistant
Ofloxacin	U	≤1	Sensitive

Dose and usage are for adults. Group A (drug of first choice): priority selection; Group B (drug of first choice): selection of drug resistance or nonuse in Group A; Group C: selection of drug resistance or nonuse in Group A and B; A supplement to the urethra; Group O: other drugs. *Kluyvera ascorbata* with the ability to produce extended spectrum beta-lactamases is resistant to penicillins, first-generation to third-generation cephalosporins, and most beta-lactams, even with a sensitive result in vitro.
